# Indonesian Adolescents’ Perceptions of Front-of-Package Labels on Packaged Food and Drinks

**DOI:** 10.1016/j.cdnut.2025.104586

**Published:** 2025-03-08

**Authors:** Wendy Gonzalez, Eny Kurnia Sari, Aang Sutrisna, Zineb Félix, Nabila Ernada, Adhika D Dibyareswati, Lindsey Smith Taillie

**Affiliations:** 1 The Global Alliance for Improved Nutrition, Geneva, Switzerland; 2 Médecins Sans Frontières, Geneva, Switzerland; 3 QUN Studios; 4 Department of Nutrition, Carolina Population Center, University of North Carolina, Chapel Hill, Chapel Hill, NC, United States; e The Global Alliance for Improved Nutrition, Jakarta, Indonesia; f Independent consultant, Jakarta, Indonesia

**Keywords:** food purchasing, dietary patterns, ultraprocessed foods, nutrition transition, food policy

## Abstract

**Background:**

Unhealthy snacking among Indonesian adolescents is common and contributes to rising rates of overweight and obesity. With the growing availability and marketing of ultraprocessed snacks, front-of-package labels (FOPLs) have emerged as a promising tool to help adolescents make healthier snack purchases.

**Objectives:**

This study aims to explore Indonesian adolescents’ drivers of snack purchase, perceptions of different FOPLs, and views on design features that could influence the impact of FOPLs.

**Methods:**

We employed a mixed-method design, incorporating both focus group discussions (FDGs) and an online survey. Eight FDGs were conducted with 46 participants aged 12–18, of different socioeconomic status from Jakarta metropolitan areas. Three FOPLs, a warning label, traffic light label (TLL), and “healthier choice” label, were tested both independently and as part of snack packages. The Pan American Health Organization nutrient profile and the UK nutrient profile were used for the warning label and TLL, respectively. Participants completed a demographics questionnaire, then an online assessment and discussion that encompassed food purchasing decisions, reactions to each of the FOPLs, comparison of FOPLs, and comparing alternative label designs for each FOPL type. Thematic analysis and the Likert scale were used for quantitative and qualitative analysis, respectively.

**Results:**

Although adolescents preferred the TLL, finding them informative, attractive, and believable, the information conveyed by the TLL was not well understood, particularly by participants with low socioeconomic status. In contrast, although warning labels were less well-liked, they were better understood, grabbed their attention, and were perceived as most likely to discourage them from buying unhealthy foods. The healthier choice label was perceived as least likely to discourage purchases of unhealthy foods.

**Conclusions:**

Although TLLs are best liked among Indonesian adolescents, warning labels are perceived as most likely to help adolescents identify unhealthy foods and discourage their purchases.

## Introduction

In Indonesia, a significant public health issue among adolescents is the high prevalence of undernutrition and micronutrient deficiencies coupled by rapidly increasing rates of overweight and obesity [1,2]. Adolescence is a particularly vulnerable period of biological development, characterized by rapid growth coupled with increased demands for nutrients and energy [3]. Poor nutrition during this period can impact adolescents’ growth and lead to negative consequences for health in adulthood [4]. Unhealthy dietary behaviors are a cause for concern, particularly as dietary preferences and behaviors established early in life tend to persist into adulthood [5].

At the same time as they undergo rapid physical changes, adolescents in Indonesia are also experiencing rapid changes in their food environment. As in other low- and middle-income countries, increasing incomes, urbanization, and the expansion of the processed food industry have increased access to ultraprocessed foods, including sugar-sweetened beverages and other industrially produced products that tend to be low in beneficial nutrients and high nutrients of concern such as sugar, sodium, and saturated fat [6]. Increased autonomy and agency along with the strong desire for belonging creates circumstances that foster a rise in unhealthy dietary behaviors [7]. Indonesian adolescents practice frequent snacking and increased consumption of food away-from home, with high intakes of fast food, fried foods and snacks, soft drinks, and ultraprocessed food, and overall poor dietary diversity [[8], [9], [10]]. A recent qualitative study of Indonesian adolescents found that they were increasingly purchasing their own snacks and meals, at schools, at work, and at vendors [9].

In the context of a rapidly changing food environment characterized by high levels of ultraprocessed food, a high prevalence of marketing of these products to children and adolescents, and the widespread use of price promotions and other strategies to promote these products, front-of-package labels (FOPLs) have emerged as a promising strategy to help consumers make healthier food choices [11,12]. Interpretive FOPLs, which provide a qualitative assessment or recommendation about the product in addition to summarizing its nutrition information, are especially promising. Among the most commonly tested label types are traffic light labels (TLL), which include color-coded and numeric information about specific nutrients of concern; warning labels, which indicate that a product is high in nutrients of concern (for example, sugar, saturated fat, salt, trans fat); and positive "healthy" logos, which indicate that a product is “healthy” according to some prespecified set of nutrient criteria (for example, meeting a minimum value for dietary fiber or not exceeding the maximum values for energy, saturated fat) [13,14]. Of these label types, the warning label is perhaps the best studied to date. Several recent systematic reviews, primarily based on experimental evidence, have shown that warnings reduced selection of unhealthy food and drinks by 26%–36% [15,16]. In addition, evidence from Chile, the first country to implement a system of warning labels, found that the labels decreased purchases of unhealthy foods [17] and changed mothers’ and children’s perceptions about these products [18]. In contrast, there are limited real-world data about the effectiveness of other common interpretive FOPLs, such as positive icons or TLL [7]. Real-world evidence on TLL systems has been mixed, with one study from the UK finding a sizable reduction in calories purchased linked to the traffic light policy [19], whereas another study found no association with purchases [20]. With regard to positive logos, some evidence suggests that they are linked to product reformulation [[21], [22], [23]]. Experimental evidence is mixed, but generally finds that positive logos are less effective than negative labels at influencing consumer perceptions, reactions, and choices [24,25]. One study found that a positive logo was linked to increased market share of logo-eligible products [26], but otherwise there are little real-world data on their influence on consumer purchase. Furthermore, little is known about how consumer characteristics influence response to FOPLs. A recent systematic review found that consumers with low socioeconomic status (SES) may benefit less from FOPLs compared with higher SES groups [27].

Despite the growing evidence about FOPLs, a major gap in the literature is the lack of studies examining how adolescents perceive, understand, and use FOPL. There are unique considerations to adolescent development that affect how they make choices about food and thus their response to FOPLs. First, adolescents may be less motivated by nutritional or health concerns than other age groups, especially boys [28]. A recent qualitative study of 18 countries found that adolescents’ food choices were influenced by family, social media, and the internet, followed by television, friends, and marketing (for example, branding, advertising, and celebrity endorsements) [29]. Thus, it is important to understand whether adolescents’ snack purchasing decisions would be influenced by FOPL, considering that the basic premise of FOPL is to help consumers better identify which products are more or less healthy [13]. In addition, adolescence is a period of increasing autonomy, including the desire to separate from one’s family and the influence of one’s parents. Healthy foods, including fruits and vegetables and home-cooked meals, may be associated with parents and home life, whereas unhealthy foods may be associated with pleasure, socialization, and independence [7]. These factors may influence how adolescents react to different FOPLs that communicate healthfulness. Thus, a better understanding of what drives adolescent food choice and response to different FOPL types in the Indonesian context is needed to identify an effective FOPL for this population.

Finally, the evidence highlights the importance of different design features of FOPLS for ensuring consumers’ understanding of labels. A recent study in South Africa focused on warning labels found that consumers preferred black triangles on a white background, the words “high in” and warning” in bold and uppercase text, an exclamation mark, and an icon depicting the excessive nutrient [30]. A similar study from the United States found that the use of marker words and exclamation points increased perceived effectiveness of warnings [31]. Less is known about the impact of design features for TLLs or positive icons. Given adolescents’ unique psychological profile, characterized by ongoing brain development, identity exploration, and heightened emotional response [32,33], it is important to understand which design features are perceived as most effective.

The objective of this study was to explore Indonesian adolescents’ *1*) factors influencing their snack and beverage purchasing decisions; *2*) perceptions of different FOPLs; and *3*) views on different design features that could influence the impact of FOPLs. A secondary objective was to examine whether these factors differed based on gender, age, and SES.

## Methods

We employed a mixed-method design, incorporating both focus group discussions (FDGs) and an online survey. We conducted 8 focus groups between 2 and 4 June, 2021. The total sample comprised 46 adolescents aged 12–18 y from the Jakarta metropolitan region, Indonesia’s most populous area. Each focus group had 4–7 participants and lasted between 1.5 h and 2 h. Focus groups were stratified by gender and age group (12–15 y and 16–18 y groups). Gender identity and gender were collected as part of the initial screening questionnaire, where participants reported their gender. Total sample size was determined based on prior studies of similar design [34], aiming to reach theoretical saturation.

We purposively recruited participants from different SES. Two groups in South Jakarta represented high-middle SES, 4 groups in East and Central Jakarta represented middle-low SES, and 2 groups in West Jakarta and Tangerang represented low SES. This distribution allowed for a broad range of participant perspectives across varying socioeconomic backgrounds (Table 1). Initially, SES was determined based on the location and type of school attended (government-subsidized, private, vocational, and international school). We then collected additional information using variables from the National Socioeconomic Survey, including parent’s education, parent’s occupation, weekly pocket money, number of vehicles (cars and motorcycles), and household floor and wall type, to further assess and confirm SES [35].

**TABLE 1:** Stratification of focus group participants by location and SES.

	Groups 1 and 2 (*n* = 13)	Groups 3 and 4 (*n* = 12)	Groups 5 and 6 (*n* = 10)	Groups 7 and 8 (*n* = 11)
Location	South Jakarta	East Jakarta	Central Jakarta	West Jakarta and Tangerang
SES	High middle	Middle low	Middle low	Low

Abbreviation: SES, socioeconomic status.

Adolescents were recruited through digital ads, peer-to-peer recruitment, and in-person recruitment in selected areas of West Jakarta, East Jakarta, South Jakarta, and Depok. For digital ads and peer-to-peer recruitment, interested individuals were directed to contact the research team to indicate their willingness to participate in the study and verify their eligibility to participate. Independent of the recruitment procedure, participants submitted the signed consent form from their guardians or parents to the study team and met with them in-person to review and complete the informed consent process. Written informed consent was also obtained from the participants before participation. The study was approved by the Ethical Committees of University Atmajaya, Jakarta with registration number 0030S/III/LPPM-PM.05.05/05/2021.

### Stimuli

#### Label design

**FIGURE 1:**
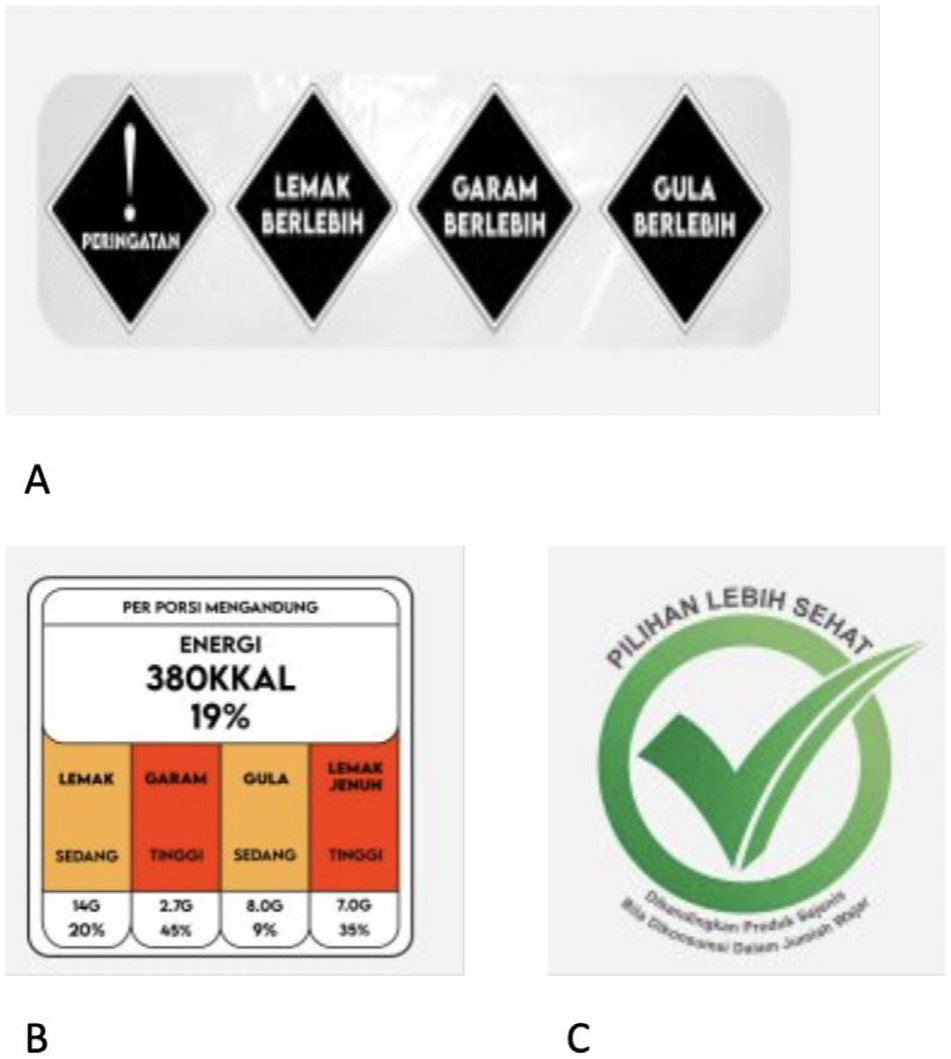
Images of front-of-package labels (FOPLs) tested in this study. (A) Warning label, (B) traffic light label, and (C) healthy check label.

Three FOPLs were selected for testing: a TLL, warning label design, and a healthier choice logo. The “Healthier Choice” logo was selected because it is already displayed on a subset of product categories in Indonesia [32], whereas the TLL and warning labels were chosen because they are used as FOPLs in other countries [17,20] and because they are under consideration by the Indonesian government for implementation in Indonesia. Images of the FOPLs are depicted in Figure 1.

(1) The warning label’s design was based on the warning label used in South Africa. It comprised 1 black diamond-shaped warning on a white background (with the text “WARNING” and an exclamation point) and ≤4 similar diamond-shaped warnings (with text, HIGH IN SUGAR, HIGH IN SODIUM, HIGH IN FAT or HIGH IN SATURATED FAT), written in Indonesian.
(2) The TLL’s design was based on the voluntary system used in the United Kingdom [36]. The label showcased information on a product’s energy and amount of fat, saturated fat, total sugar, and salt, using color codes—red, yellow, or green—to signal the nutrient content as high, medium, or low, respectively.
(3) The Healthier Choice label’s design was based on the icon used for selected instant pasta and noodles, and ready-to-drink beverages in Indonesia. The icon comprises an encircled green tick sign with the words “HEALTHIER CHOICE” written on top, and “Compared to similar products when consumed in fair quantities” at the bottom of the circle [32].

For each label, we tested a set of alternate designs developed by the study team (Table 2). Participants were queried about a range of design elements, such as font size, color of label and "holding strap" (that is, a design element used to embed the logo, for improving aesthetic and visibility), and the shape.

**TABLE 2:**
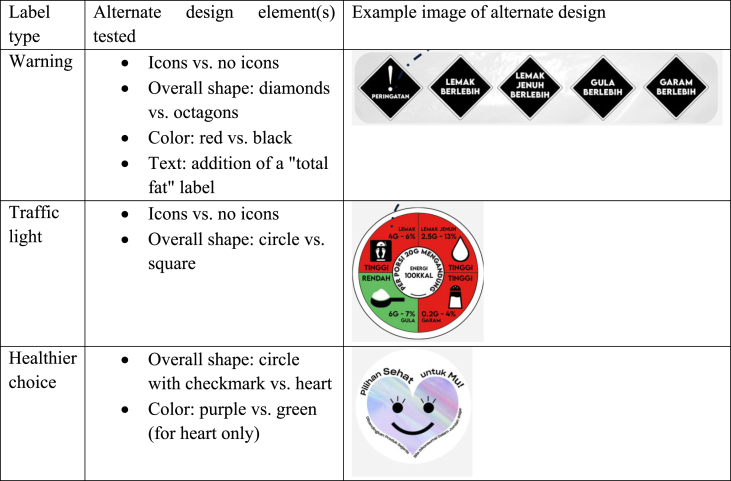
Design elements tested for each label type.

To enhance realism, we presented the labels within digital and physical mock-ups resembling real Indonesian snacks commonly preferred by adolescents. The selection of snacks was guided by interviews with 4 adolescents, 2 boys and 2 girls, living in Jakarta. This selection was cross verified with the project’s dataset detailing frequently consumed packaged foods by Indonesian adolescents (data not published). The product categories shown to participants were consistent across each type of label and encompassed biscuits, ready-to-drink beverages, chips or crisps, and instant noodles.

#### Nutrient profile models

Different nutrient profile systems were used for each label type to determine the relevant information to display for each product. For instance, for the warning label, this encompassed determining the number and type of warnings to be included, and for the TLL, it involved deciding the color and numeric information for each nutrient. We used the Pan American Health Organization (PAHO) nutrient profile [37] and the United Kingdom nutrient profile [36] for the warning label and TLL, respectively. For the healthier choice logo, we used the nutrient profile established in the Indonesian Badan Pengawas Obat dan Makanan (BPOM) norm 22 [32]. In each case, the nutrient profile model was applied to the real nutritional information of each product. As an exception, chips and biscuits for the healthier choice icon were rated using the PAHO nutrient profile, as BPOM norm 22 did not include cutoffs for these product categories during the study period.

### Procedures

We initially pretested the quantitative data collection tool and conducted online cognitive testing of the FGD guide with a sample of 6 adolescents. The findings from these activities were used to refine and finalize the study instruments.

Trained moderators from a market research company used a semistructured FDG guide prepared by the research team to collect data. First, participants completed a demographics questionnaire, including information on age, gender, school level, school type, parent’s education, and parent’s occupation. Then, they alternated between completing an online questionnaire and participating in a moderated discussion. Both the questionnaire assessment and the moderated discussion guide were adapted from instruments used in previous studies [38], and they comprised 4 sections: *1*) food purchasing decisions; *2*) reactions to each of the FOPLs; *3*) comparison of FOPLs (for example, the TLL compared with the warning label compared with the healthier choice icon); and *4*) comparing alternative label designs for each FOPL type. During each section, the moderator displayed product mock-ups with the relevant label types for participants to observe during the ratings assessment and discussion. The full discussion guide is presented in Supplemental material 1.

In the food purchasing decisions section, the group explored *1*) the most important factors influencing their purchasing decision; *2*) use of back-of-package nutrition information; *3*) use of front-of-package nutrition information; *4*) use of nutrition and health claims; *5*) appeal of product packaging and marketing elements (for example, liking of cartoons or celebrities); *6*) consideration of price, healthfulness, and “coolness”; and *7*) meaning and potential use of government endorsement of front-of-package nutrition labels. In the section on reactions to FOPLs, participants explored *1*) visibility and memorability; *2*) comprehensibility; *3*) perceived effectiveness on attitudes toward products and purchasing behavior; and *4*) design elements of the label (for example, which aspects of the FOPL were most memorable).

In the comparative ratings section, participants were shown all 3 FOPLs and asked whether 1 label stood out and if so, why. Participants also discussed which label would most help them identify unhealthy foods and would most discourage them from buying unhealthy foods. Finally, in the comparative alternative label designs, participants were shown alternative designs of the FOPLs and discussed their *1*) visibility and memorability; *2*) comprehensibility; *3*) perceived effectiveness on attitudes toward products and purchasing behavior; and *4*) whether the alternations in the design influenced their intention to consume a product.

### Questionnaire measures

The Typeform platform [39], an online tool for creating surveys, was used to administer the questionnaire. Participants were not required to download any software, as they could access the survey directly via a web link on their phones.

Using the pretested questionnaire, participants were asked to report their most frequently consumed snacks and beverages and to circle and rank the top 5 factors that were most important when making up their mind about a new food or drink to examine factors influencing food purchases. Responses included brand, price, presence of characters, promotions/gifts/discounts, flavor/taste, nutritional information, familiarity, convenience, recommendations by health professionals or institutions (for example, ministry of health), advertisements, family preferences or requests, or nutrition/health claims.

Participants were then shown product mock-ups with particular label, and asked to rate how much they agreed or disagreed that the label was *1*) easy to see; *2*) memorable; *3*) believable; *4*) made them stop and think; *5*) relevant; *6*) helped them know if foods/drinks are unhealthy; *7*) made them concerned about purchasing unhealthy foods/drinks; *8*) made them less likely to purchase unhealthy foods/drinks. Response options included strongly disagree, somewhat disagree, neither disagree nor agree, somewhat agree, or strongly agree. Actual wording for items and responses are shown in Supplemental material 2. The sequence in which the labels were presented differed across FGD.

After seeing all 3 labels, participants were asked to select which of the 3 options: *1*) was most likely to grab their attention; *2*) which one would help them tell which foods and drinks were unhealthy; and *3*) which one was most likely to avoid purchasing unhealthy foods or drinks (actual wording shown in Supplemental material 3). Finally, in the comparative alternative label designs, we asked participants to observe 3 alternative designs for each FOPL and select their preferred option.

All discussions were conducted in Indonesian, recorded and transcribed verbatim, and translated into English for analysis.

### Analysis

Descriptive statistics on the sociodemographic characteristics of the sample were tabulated.

For the qualitative analysis, we used a thematic analysis approach [40,41]. Initial codes were defined based on our analytical interest and previous research regarding food purchasing behaviors and reactions to FOPLs [30,42]. Some of the deductive codes served as main themes, some of which aligned with specific interview questions [30,42]. After coding 3 different transcripts using Nvivo software, 3 analysts used inductive analysis to identify new codes and subthemes. All transcripts were reviewed collectively and through discussions by the research team, the codebook was refined and updated during the data analysis phase. The deductive approach ensured that relevant themes from prior work were captured, whereas the inductive process allowed for the emergence of new insights from the data.

For the quantitative assessments, the Likert scale used for each item (5–1, strongly agree, somewhat agree, neither agree nor disagree, somewhat disagree, and strongly disagree) was recorded to agree (5 and 4), neutral (3), or disagree (1 and 2). For each item, the percentage of adolescents responding “strongly agree” or “somewhat agree” was calculated. We used descriptive statistics to analyze the ranking for the top 5 factors influencing snack purchasing decisions. We calculated the frequency of each factor being ranked within the top 5, and this was then used to generate overall rankings.

## Results

Table 3 presents the sociodemographic characteristics of the study participants. The sample included 23 boys and 23 girls, with 23 participants between the ages of 12 and 14, and 23 participants between the ages of 15 and 18. The majority of participants’ mothers were housewives, with public employment and entrepreneurship being the next most common maternal occupation. Approximately half the sample’s mothers had completed high school, whereas 32% completed college or graduate school. In terms of paternal occupation, daily worker and private worker were the most common occupations, followed by entrepreneur and other professional. Fathers were more educated, with 33% having completed college and 43% holding a master’s degree or higher. Regarding household assets, the vast majority (87%) of participants’ families owned a motorbike, whereas 57% owned a car.

**TABLE 3:** Sociodemographic characteristics of the sample.

	*N* (%)
Age group	
12–15	23 (50)
16–18	23 (50)
Gender	
Boy	23 (50)
Girl	23 (50)
School level	
Senior	25 (54)
Junior	21 (46)
School type	
Public	18 (39)
Private	9 (20)
International	7 (15)
Vocational	7 (15)
Other	4 (9)
Missing	1 (2)
Maternal occupation	
Housewife	26 (57)
Daily worker	2 (4)
Public worker	8 (17)
Civil servant	1 (2)
Entrepreneur	7 (15)
Doctor and other professional	1 (2)
Missing	1 (2)
Paternal occupation	
Daily worker	13 (28)
Private worker	15 (33)
Public worker	4 (9)
Entrepreneur	9 (20)
Doctor and other professional	5 (11)
Maternal highest education	
Elementary	3 (7)
Junior high	3 (7)
Senior high	23 (50)
Bachelor’s degree	7 (15)
Master’s degree or higher	8 (17)
Missing	1 (2)
Paternal highest education	
Elementary	4 (9)
Junior high	1 (2)
Senior high	4 (9)
Bachelor’s degree	15 (33)
Master’s degree or higher	20 (43)
Missing	1 (2)
Assets: family owns car	26 (57)
Assets: family owns motorbike	40 (87)
Adolescent weekly pocket money	
<IDR 50,000 (<$3 USD)	16 (35)
IDR 50,000–100,000 ($3–7 USD)	18 (39)
IDR 100,000–250,000 ($7–17 USD)	9 (20)
IDR 250,000–350,000 ($17–24 USD)	2 (4)
>IDR 350,000 (>$35, USD)	1 (2)

Daily worker (for example, online driver, security, and laundry); private worker (advertising, general manager, and architecture).

Abbreviation: IDR, Indonesian Rupiah

Most participants reported frequently purchasing snacks. The most frequently consumed snacks and beverages included puff snacks made out of corn, rice, or wheat, potato chips, bread, biscuits, flavored dairy products, and ready-to-drink teas.

Table 4 depicts the 9 themes and 34 subthemes identified in the qualitative analysis.

**TABLE 4:** Themes and subthemes from qualitative discussions.

Section	Theme	Subtheme	Example
Food purchasing decisions	Product packaging	Cartoon characters Nutrition information (front and back) Ingredients Health/nutrition claims Attractiveness	“Cute characters, interesting text.” (Girl, FGD8, age 12–14) “If it has an interesting color, or an interesting character.” (Boy, FGD7, age 14–18) “I like darker colors better. Like brown. I like things because that’s my favorite color too right, so like oh that’s nice. It’s cute.” (Girl, FGD6, age 15–18)
	Product characteristics	Brand/name Flavor Price Discount or promotion Advertising (not related to packaging) Endorsement	“Yeah, the brand. I prefer if it has a brand.” (Boy, FGD7, age 14–18) “It really comes down to flavor. If for example one is called unhealthy but the unhealthy snack tastes better, (…) I don't really care.” (Boy, FGD5, age 16–17) “For me, yeah, also from advertisements. Like if there’s something new.” (Girl, FGD6, age 15–18) “Idols, right? KPop.” (Girl, FGD8, age 12–14)
	Product perceptions	Taste Familiarity Convenience Coolness Healthfulness Purchased by friends or family	“Because if the price is cheap, but the snack doesn't taste good then there’s no use in buying it.” (Girl, FGD8, age 12–14) “That tastes good.” (Boy, FGD7, age 14–18) “If the product [...] if I'm told that the product is healthy, then I'll buy it. But if for example if I'm told it’s unhealthy, then I'll buy it but not often.” (Girl, FGD4, age 12–15) “…then he says ‘Man, try this man. This is so good, man.’ Then I'd try it, and it’s good. Or like if it’s something that I'm about to take, to purchase, but then he says ‘Don't get that man. That’s not good.’ Always, things like that.” (Boy, FGD5, age 16–17)
FOPL reactions	Visibility and memorability	Visibility Memorability Ability to recall the label	“Because it’s easy to remember and it’s very significant. If you see it, you know it’s healthier. But it’s harder to spot, really hard to spot.” (Boy, FGD1, age 16–18) “Yes, the size is too small... I wouldn't notice it if I buy the product. It’s not noticeable.” (Girl, FGD2, age 15–17)
	Comprehension	Ability to understand the purpose of the label Overall understanding of label’s meaning (what it says about the product)	“Maybe... so it’s like a warning to buyers. Maybe the buyer can... suppose he or she can't eat high-sugar foods.... that means the food could affect them, so it’s like, ‘Oh I see, the sugar is high, it I shouldn't buy it.’ So it’s one thing that the buyer could consider.” (Girl, FGD2, age 15–17)
	Other label reactions	Believability Cultural appropriateness Target population of the label	“Believe it, because the label comes with the product.” (Girl, FGD4, age 12–15) “If it’s from the National Agency of Drug and Food Control then I trust it.” (Boy, FGD7, age 14–18) “Overall, that [...] is gonna promote healthy eating, I feel like right now, a lot of people are not necessarily informed about healthy eating at all. So that’s the reason why they don't really care, you know what I'm saying? But if you teach, like, younger [...] this label, it will inform children to like, make better decisions. and the new generation will adapt easily to that.” (Boy, FGD1, age 16–18)
	Perceived effectiveness	Potential effect on product attitudes Potential effect on intentions to purchase Perceived benefits and harms Likelihood to use	“People will think twice before choosing this product...” (Girl, FGD4, age 12–15) “Yes because I'll think, ‘Oh this is healthy’, I'll buy it” (Boy, FGD3, age 14–15)
FOPL comparison	FOPL comparison	Selection of which FOPL was perceived as most effective	“Because the first one, we can decide for ourselves whether something is healthy or not from the percentages. With the second one, it already tells you ‘This one isn't good, it’s not healthy.’” (Girl, FGD4, age 12–15) “Yes, and also like, the first one would tell us, what is it, this is high sugar, this is low sugar, there’s more detail given for us to make a choice.” (Girl, FGD4, age 12–15) “Because there’s a warning that literally reads warning. I don't even want to bother, like the other one, like the first one you can consider like, should I buy it, should I buy it. But this one, I won't even bother. Because it already says warning and there are no grams for things, so I'd immediately assume that it has too much fats. It’s too scary. The color too.” (Girl, FGD6, age 15–18)
Alternative design comparison	Label or design preference	Attractiveness and memorability Informational Perceived effect on purchasing decisions Overall preference	“But the fact that it’s a heart is, perhaps, more eye-catching, than just a checkmark. It’s just that this heart shape is ugly.” (Boy, FGD1, age 16–18) “It’s nicer to look at. The design is not tilted and the top and bottom of the label is clean and organized.” (Girl, FGD2, age 15–17) “Okay. Because there is a picture... So it’s more... understandable and it makes you not want to buy it.” (Girl, FGD4, age 12–15)

Abbreviations: FGD, focus group discussion; FOPL, front-of-package label.

### Main factors influencing snack purchasing decisions

**FIGURE 2:**
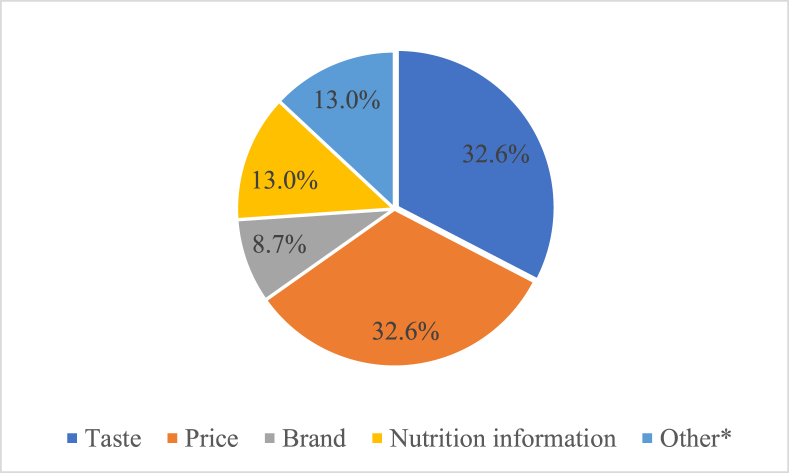
Top-rank factors driving food purchasing decisions (percent selecting factor). ∗Other included presence of characters (4.3%), familiarity (4.3%), and liked or requested by family members (4.3%).

Figure 2 shows the top factors influencing snack purchasing decisions reported via the forms. All groups mentioned price and taste as an important determinant influencing food purchases; a third of participants reported them as top factors. Nutrition information and brand were less important (reported as the top factor by 13% and 9%, respectively), as were other factors, such as the presence of characters in the package, familiarity with the product, and whether the product was liked or requested by family members (13% total).

During the discussion, participants reiterated the importance of taste and price as primary factors influencing purchasing decisions and highlighted the importance of obtaining good value for money for their purchase. For instance, they reported not seeing the value of purchasing expensive snacks, when cheaper options also have good taste and quality; or buying snacks with a higher price tag and smaller quantities that might not be filling.

“Even expensive snacks do not always taste good, so why buy expensive snacks when there are cheap ones that also taste good?” (13-y-old girl, FGD 8)

“For example, a big bag of Lays is a fourth of chips and the rest is air. So the food itself is not enough.” (14-y-old boy, FGD3)

Some participants mentioned during the discussion reading the nutritional information for selecting snacks. Those who did were generally more health conscious, often because of reasons such as practicing sports or following a diet. Few adolescents understood the meaning of the nutrition information on the nutrition facts panel, or how to use this to determine whether a product was good for their health. One of the participants who read the nutrition labels mentioned:

“I mostly pay attention to carbohydrate, protein, as well as saturated fat and the total. […] Like you just want to know the amount but you don't know if it’s high or low. I trust nutritionists more. I was told to pay attention to labels when I shop for snacks.” (17-y-old girl, FDG 2).

Additional factors discussed that influenced purchasing decisions encompassed the presence of Halal labels (indicating compliance with Islamic dietary laws), expiration date, and recommendations from friends.

“For example, if [name] has already bought it, he'd say, "Hey, man, give it a try. It’s good, man." So I've tried it, and it’s good. But, for instance, maybe I've already taken it or was about to buy it, and then he says, "Hey, man, not that one, man. It’s not good." It’s like that, definitely like that.” (16-y-old boy, FGD 5)

Mood and cravings and the budget available for purchase were also noted as influential elements in their decision-making process. Within 1 group, a female participant highlighted the eco-friendliness of products as a factor, favoring those with paper straws or no straws.

### Product packaging and marketing elements

Participants paid attention to packaging and were attracted to products that were considered “cool” or aesthetically pleasing. Design preferences varied by age group, although attractive package colors were important to all. Older adolescents were more attracted to minimalist designs and designs that implied luxury or exclusivity, whereas younger children were more attracted to packages with characters, sports or celebrities, or games.

“For me, it’s more about the colors maybe, [...] So if the packaging is black or gold, it feels more exclusive.” (16-y old boy, FGD1)

### Perceived healthiness of snacks

Only a few participants were able to articulate the distinction between healthy and unhealthy products. Unhealthy products were identified based on various factors, including their ingredients, flavors, nutritional content, food safety, and freshness. Certain participants deemed products with elevated levels of calories, sugar, sodium, monosodium glutamate, preservatives, oils, or spiciness as unhealthy. However, others considered products that were stale, nearing or past their expiration dates, or obtained from unhygienic sources to be unhealthy. A subset of participants also recognized the link between consuming foods high in sugar or salt and the development of chronic conditions like hypertension or diabetes.

Conversely, participants characterized healthy products as those containing specific ingredients like seaweed or milk, having particular nutritional qualities such as higher protein or fiber content, or belonging to specific food categories like bread. Additionally, perceptions of healthfulness were influenced by attributes like being Halal, not being overly tasty or filling, or being on the more expensive side. Furthermore, participants’ views on healthiness were shaped by advertising and packaging, encompassing factors like product colors, with green being associated with healthiness, and nutritional claims. One participant mentioned:

“It’s because just from the advertising you’re already convinced. Like, you want to have a snack but you don’t want to gain weight, so you snack on a FitBar. And then from the packaging too, from, what, the packaging makes this impression that it’s super healthy. The color is green. And there’s, what is it, the phrase ‘low-fat’”. (17-y-old boy, FGD 5)

#### Reactions to FOPLs

Table 5 shows the quantitative ratings on reactions to FOPLs. Overall, most participants considered that each of the 3 FOPL types was easy to see, memorable, and believable. They particularly took note of the warning label’s black color, its simple design, exclamation point, and the word “warning,” which elicited a sense of danger, fear, or threat in some individuals.

**TABLE 5:** Participant reactions to FOPLs: percent who agreed or strongly agreed with each statement (*n* = 46).

The label is…	Warning (%)	Traffic light (%)	Healthy icon (%)
Easy to see	83	83	72
Memorable	67	67	76
Believable	65	76	74
Makes me stop and think	67	57	52
Is relevant	67	67	59
Helps me identify unhealthy food	87	80	72
Makes me concerned about purchasing unhealthy food	52	37	37
Discourages unhealthy purchases	35	35	37
Decreases intentions to purchase unhealthy food	28	26	28

Exact item wording and responses can be found in Supplemental material 2.

Abbreviation: FOPLs, front-of-package labels.

In general, participants were drawn to the TLL’s design, described as catchy, “cool,” and memorable. Although the Healthier Choice label received the highest rating for memorability (76% of participants), the ensuing discussion revealed that participants viewed this label as the least visible, difficult to notice, uninteresting, or unattractive. One participant elaborated on how the green label blended in with the product’s packaging:

“The color of the packaging is sometimes green and the color of the checkmark is also green, so there’s not enough contrast.” (14-year-old girl, FDG 3)

The TLL and healthier icons were the most believable labels (76% and 74%, respectively). Elements of the TLL that made it believable and trustworthy included its colors, design, and display of numeric information. Some participants considered the warning label too frightening, or aggressive to be believable; others found it misleading, as they did not believe that any food product could pose a danger to adolescent health. Interestingly, participants suggested adding a government endorsement (for example, the Ministry of Health or National Agency of Drug and Food Control) to make the warning label more believable. Furthermore, some deemed the Healthier Choice label unbelievable when displayed on packages of products perceived as unhealthy, such as sweetened milk, instant noodles, crips or chips.

Most participants considered that the warning label made them stop and think (67%), helped them identify unhealthy food (87%), and made them concerned about purchasing unhealthy food (52%). They understood the warning label and its purpose, describing it as a means to inform consumers that a product contains high amounts of nutrients of concern (for example, sugar, saturated fat, and fat). Participants recognized the label as a “warning” intended to make them stop and think, though it would not necessarily discourage purchases. Warning labels would help them identify whether a product was unhealthy or not.

Although most participants considered that the TLL would make them stop and think (57%) and helped them identify unhealthy food (80%), few believed the label would make them concerned about purchasing unhealthy food (37%). Most understood the meaning of the colors of the TLL, and some considered that the label would help them determine if a product was healthy or unhealthy. However, some did not understand the meaning of the colors and/or found the numeric information provided in the label to be confusing.

Green is like, green is still safe. Yellow is fine, but not too often. If it’s red you shouldn’t have it often (boy, 16, public school).

I don’t understand what the percentages mean either (…). It’s not really important, and I’m too lazy to read. The important thing is the warning (Boy, 15, public school).

Overall, few participants agreed that any of the 3 label types would make them not want to buy unhealthy foods (35%–37% of participants) or discourage them from purchasing unhealthy foods (26%–28% of participants). Boys were less inclined to report that any label would be effective at changing their behavior. They perceived the labels as being for someone else (for example, possibly peers, but only if they were on a diet or conscious about their health; parents would also be receptive to the label, and young children who are open to learning about healthy eating). Girls, however, seemed more open to the influence of the labels in their purchasing behaviors.

Opinions about the labels’ influence on their snack purchasing behaviors were mixed. Warning labels appeared to exert a slightly stronger influence compared with other labels. Their clear and simple message and alarming connotation prompted a call to action.

“Because people who don’t understand much can immediately get to the point. The warning is good for people who don’t want the hassle.” (15-year-old girl, FGD3)

Some individuals found this label to be "frustrating" because it restricted their choices and felt like it was "telling them what to do." These individuals expressed a desire to rebel against such labels by purchasing the product. One participant mentioned its resemblance to the cigarette warning labels and explained:

“It’s like, you know… there’s a [...] cigarettes have warning label, right? I think it’s kind of like that. It won't necessarily, completely stop people from buying it. But at least it'll make people think and consider like, oh, this is what I'm getting myself into. [...] a little bit guilty.” (18-y-old boy, FGD1)

Some participants appreciated the positive nature of the “Healthier Choice” logo, noting that it could help them identify what products were healthy, particularly for those wishing to have a healthier lifestyle. Although some considered that this label would help them choose healthier products, most characterized the label as not noticeable, too simplistic, and without any ability to make them stop and think or influence their behavior.

#### FOPL design

Participants commented on each FOPL’s design as well as compared it with other versions of the same FOPL (for example, with different shapes or colors). For warning labels, the word “WARNING” and the exclamation point were the design features that stood out the most. Participants considered that the black color enabled contrast and was easy to read. However, red was better at signaling warning, but was distracting or hard to read because it blended in with the package. Participants perceived that the harsher the outline shape, the scarier it appeared compared with a rounder outline. Reactions to the use of an icon varied by SES: although lower SES participants preferred warnings with icons, higher SES described them as distracting. Some expressed that the icons helped make the label seem friendlier or less scary.

Overall, adolescents found the design of the TLL most attractive (for example, it was “cool” or “exciting”). They found the colors especially attractive and enticing, although some pointed out that they blended in with the package. Reactions to shape were mixed (some preferred circles whereas others preferred squares). Similar to the warning, reactions to icons were mixed, with low-SES participants more inclined to prefer icons whereas high-SES participants preferred more simplistic designs.

Finally, participants found the check on the “Healthier Choice” label to be too small in size, with too small font, and that although they understood that the green color signaled healthy, it blended in with packages. When comparing the check to the heart, participants felt the heart was childish, unclear in meaning, and “weird.”

#### FOPL comparison

Table 6 presents the participants’ selection of their preferred label. The largest proportion of participants chose the TLL as the most attention-grabbing (41%) and most helpful in identifying unhealthy products (67%). Meanwhile, the highest proportion of participants selected the warning label as most likely to discourage purchases of unhealthy products (46%), followed by the traffic light (41%).

**TABLE 6:** Selection of FOPL most likely to grab attention, help identify unhealthy products, or discourage purchase of unhealthy products (*n* = 46).

	Warning (%)	Traffic light (%)	Healthy icon (%)	All are the same (%)
Grabs attention	28	41	28	2
Identify unhealthy products	26	67	7	0
Discourages purchase of unhealthy products	46	41	19	2

Exact item wording and responses can be found in Supplemental material 3.

Abbreviation: FOPL, front-of-package label.

During the discussion, participants perceived the warning label as highly attention-grabbing and appreciated its simplicity. However, they found it less visually attractive as compared with the TLL. Warnings were seen as conveying a negative tone, whereas TLL were considered more positive, "friendly," educational, and informative. At the same time, participants found warnings to be clearer and easier to understand due to their simplicity, whereas the TLL was harder to understand, particularly for younger or less educated individuals. The Healthier Choice label was perceived as the least useful because it exclusively referred to healthy products. Although a few participants noted that the warning label evoked a sense of rebellion, it was the label most commonly mentioned as likely to decrease purchases of unhealthy foods due to its simplicity and negative connotation.

## Discussion

### Main findings

In this set of focus groups of Indonesian adolescents aged 12–18 y, adolescents revealed that price and taste, rather than nutrition considerations, were the primary factors influencing food purchasing decisions. When evaluating FOPLs, adolescents expressed a preference for the TLL, finding them more attractive and believable, and viewing them as more informative. However, despite liking the information conveyed by TLL, a major drawback was that the information was not well understood, particularly by participants with low SES, making it unlikely to influence their purchasing behavior. In contrast, adolescents reported that warning labels effectively grabbed their attention, were well understood, and were most likely to discourage them from buying unhealthy foods, representing crucial steps along the conceptual pathway to behavioral change. Nevertheless, warnings were the least favored and believable label. Finally, although adolescents liked the “Healthier Choice” label, appreciating its positive signaling rather than a negative one, it was the least noticeable FOPL. The “Healthier Choice” label was perceived as the least likely to discourage purchases of unhealthy packaged foods.

#### Main factors influencing snack purchasing decisions and implications for reactions to FOPL

Price, taste, and value for money consistently emerged as top factors influencing snack choice, consistent with previous research on both adolescents and adults [7,43]. Nutrition information was not a top driver of choices, and adolescents were not likely to use or understand existing nutrition labels. The low rate of nutrition label use was anticipated because of the lack of salience of nutrition information in the package and the absence of guidance on how to interpret it. Interpretive FOPLs can help address this issue by capturing adolescents’ attention to nutrition information and offering guidance on the subsequent purchasing decision (for example, recommendations or warnings) [13].

To complement FOPLs, interventions could leverage the key factors influencing the purchasing of snacks, such as price and value for money. For example, price subsidies on healthier snacks or incentives for retailers to promote healthier products could align with FOPL strategies, making healthier options more affordable and attractive. Additionally, targeted interventions in school settings could focus on reducing the cost of healthier snacks while increasing their appeal to adolescents [44]. These interventions, when paired with FOPLs, could create a more comprehensive strategy to promote healthier snack choices among young consumers [45].

Although some adolescents understood the link between nutrients like sugar or sodium and health, there was a limited understanding of the relationship between nutrition and chronic disease outcomes. Specifically, adolescents did not perceive nutrition as an important factor for them, a perception that influenced their reactions to the FOPLs. Indeed, across FOPL types, adolescents expressed the view that FOPLs were intended for “someone else,” that is, younger children, parents, or individuals concerned about nutrition or health—but not them. Some participants, particularly boys, reported that they were unlikely to pay attention to or use FOPLs, likely because of this lack of knowledge or interest in nutrition.

Previous research has found that high health consciousness increases adolescents’ responsivity to messaging about nutrition labels [46]. In addition, risk perceptions are an important predictor of health behavior in adolescents [47]. Tobacco research has demonstrated the effectiveness of mass media campaigns in changing risk perceptions [48] and driving behavioral change [49] in adolescent populations. This suggests that a potential strategy to increase receptivity to FOPL messages is to integrate tailored communication campaigns within the FOPL intervention aimed at raising awareness about the association between poor nutrition and health risks among adolescents.

Social norms, approval, and interaction were not mentioned as a top driver of food purchases. Social norms and social approval have a strong influence on specific health-related behaviors [50], and this is likely to be especially heightened during adolescence. Furthermore, social norms play an important role in food choice in the presence of peers [51]. Participants may not have explicitly mentioned social norms or interactions as a main driver of purchases as these factors might operate at a subconscious level, making them unaware of their influence on purchasing decisions. Moreover, the alterations in lifestyle brought about by the recent COVID-19 pandemic [52] likely led to a decrease in social interactions associated with snacking at the time this study was conducted.

Social interactions play a role in certain mechanisms through which labeling systems, particularly warning labels, can exert their influence. Research from tobacco labeling has shown that increased conversations about warning labels correlate with behavior change [53]. Understanding how social interactions influence the interpretation and use of FOPLs is important, especially considering that adolescents frequently encounter FOPLs in the company of their peers (for example, when purchasing snacks with their friends at the minimarket, warung, or at school). Labels that stimulate social interaction may be more likely to bring about changes in purchasing behavior [53]. However, social interactions have a limited effect if products carrying a warning label or TLL are priced more cheaply. In other words, even if there is a stigma against “unhealthy foods” (as demarcated by a warning label), adolescents still may buy them if they are cheap, because price is a top factor. Hence, delving into the relationship between FOPL and social norms and how these factors intersect with other factors like price to influence product selection merits further exploration within this demographic.

Although adolescents rated both the TLL and warning label as easy to see, easy to recall, memorable, and likely to help them identify unhealthy foods, there were several notable differences between FOPLs. The finding that participants considered TLLs more informative than warnings, but that warnings were easier to understand, is consistent with previous literature [54]. This is likely because of the complex and extensive information presented to participants on the TLL. In addition to the numeric information, which participants found confusing, TLLs can sometimes present contradictory information (for example, a product is high in one nutrient of concern but low in another), requiring consumers to analyze all the information to assess a products’ healthfulness. Indeed, previous qualitative research on labeling has found that consumers have difficulty understanding and interpreting amounts and percentages on FOPLs [55]. This poses a challenge because most consumers make decisions about food very quickly (in seconds) [56], and often make snap decisions without deeply processing the provided information [57]. In addition, although other studies have found that the color-coding (red/yellow/green) helps facilitate the interpretation of TLLs [55], this study found that certain participants were confused by the colors, suggesting that this approach may not work well with adolescent populations.

Adolescents liked the TLL the most out of all FOPL types. They found the design appealing, cool, and interesting, and they liked that the label did not tell them what to do (compared with warning labels). These results are consistent with previous literature: people do not like warning labels as much as other label types [54], yet despite this, in experiments, warnings are frequently found effective at changing behavior [16]. In this study, although the warnings were less well-liked, adolescents reported that warnings were well understood and more likely to make them stop and think and to influence their purchases. Consistently, a recent study found that among youth, mandated warning labels in Mexico and Chile had substantially higher levels of awareness, use, and understanding than the voluntary TLL in the United Kingdom [58].

Participants liked the simplicity of the TLL label and considered that the warnings provided a clear message of danger or warning that would make them stop and think. This “stopping and thinking” is important because it disrupts subconscious processes that lead to impulsive purchasing decisions or ingrained patterns of purchasing. Indeed, previous evidence from tobacco warnings has found that making people think is a key mediator on the pathway between label exposure and behavioral change [59]. The fact that warning labels also grabbed participants’ attention is critical because FOPL must be able to cut through the many other design elements on the packages that are designed to capture their attention [60], and be quickly and accurately understood. The adolescents indicated that they were attracted to many other packaging elements, so “attention-grabbing” is important for an effective FOPL design in this population. Nevertheless, a recent study showed that high level of awareness found in Chile and Mexico regarding the warning label, in contrast to lower levels related to TLL in UK likely reflected differences in mandated policies in Chile and Mexico compared with voluntary United Kingdom policies [58]. This implies that the mandatory nature of the label plays a significant role in the use of the label [54] and may have a more significant influence than the attention-grabbing aspect.

Warning labels elicited more negative emotions than other FOPLs. An increase in negative affect is consistent with the conceptual pathway for how warning labels change behavior [12] as well as theoretical and empirical research from tobacco warnings, which have found that negative affective reactions to warnings are a key trigger for eliciting behavioral change [59,61,62]. Negative emotions can help people process risk information [63] and also create more negative attitudes toward products [50]. However, some participants indicated that the labels were less believable, misleading, or extreme for characterizing a product as dangerous. One possible explanation for this was participants’ low awareness about the health harms of foods high in sugar, sodium, or saturated fat. For example, previous work in tobacco has found that when adolescents have low awareness about a product’s health harms, they find messaging about it less believable [64]. Communication campaigns about unhealthy foods and health harms could help increase knowledge and awareness, increasing the believability of warnings about unhealthy foods. Another option is to add a government endorsement on the warning label itself. According to the Elaboration Likelihood model, the source of a message can serve as a cue of message quality and influence attitudes, particularly for messages processed on the periphery of consciousness, such as food labels [57]. Indeed, in the focus groups, many adolescents noted that a government endorsement would help increase believability and trustworthiness, although empirical evidence about the effectiveness of including a government source in warnings is mixed [65]. Similar government endorsements have been used in other real-world food warning label systems, including in Chile and in Mexico, and thus warrant further investigation in Indonesia as well.

Another concern with the warning labels is reactance, which is defined as resistance to a message in response to a perceived threat to freedom [66,67]. Some older participants noted that the label was telling them what to do, which made them want to rebel against it. This response makes sense in the context of adolescent psychological development: adolescents desire independence and have a tendency to reject authority, meaning that FOPLs like warnings that are perceived to restrict their freedom are more likely to be rejected [68]. Currently, there is little understanding of the impact of nutrient warnings on reactance in adolescent populations, or how this is associated with impact on food purchasing behaviors. Evidence from tobacco in adolescent and adult populations has found that graphic warnings lead to more reactance than text-based warnings [69,70], suggesting that text-based nutrient warnings such as those used in this study may not elicit high levels of reactance. In addition, other tobacco control research suggests the reactance on behavioral change is small [61] and may even increase behavioral intentions (for example, quit attempts) in response to warnings [71]. However, reactance increases with age [68], and other FOPL elements that work well on one dimension can also increase reactance. For example, previous research has found that among adolescents, government endorsements that increase credibility can also be perceived as domineering, which can in turn increase reactance [69]. Researchers should further investigate reactance to nutrient warning labels in this population, including how the use of different types of messages (for example, implicit compared with explicit) or design features consider reduce reactance [72].

Warning labels appeared to be more likely to discourage adolescents from purchasing unhealthy packaged foods than other label types; however, adolescents overall did not seem to think any FOPL would be particularly effective at changing their behavior. This was reflected quantitatively as well as qualitatively, as participants rated all FOPLs low on perceived message effectiveness, or how much the labels make them concerned about purchasing unhealthy foods and discourage them from buying them. Perceived message effectiveness is predictive of behavior [72], which suggests that FOPLs may be less likely to affect purchasing decisions of adolescents. In addition, boys seemed less receptive to all FOPL types than girls, which may reflect underlying differences in interest in nutrition [28] or behaviors, as Indonesian girls are more responsible for food purchases and home cooking [9]. Future research is needed to understand whether FOPLs would actually impact adolescents’ food purchasing behavior in this context, as well as potential differences by gender.

Finally, the “Healthier Choice” FOPL was perceived as the overall least noticeable or effective label. One possible explanation for this is that the “Healthier Choice” contains less information than the other FOPLs tested, which is consistent with previous qualitative research finding that healthy icons were less acceptable because they do not provide information on specific ingredients or nutrients [55]. In addition, some participants were confused that the “Healthier Choice” FOPL appeared on products such as sweetened milk, which they perceived as unhealthy. Although the current Indonesian guidelines permit the use of the healthy icon on sweetened milk, this finding underscores the importance of using a strong nutrient profiling model that correctly classifies products as healthy or unhealthy, and labels them as such. The use of the “Healthier Choice” label on unhealthy products can give them a "health halo" effect, leading consumers to misjudge their nutritional quality and potentially consume even greater quantities than they otherwise would [73,74].

With regard to FOPL design, several patterns emerged across FOPL type. First, icons tended to be most useful for low-SES groups, who considered that icons enhanced comprehension, but not for high-SES groups, who preferred more simplistic designs. In addition, adolescents liked the use of colors such as red (for signaling unhealthy) or green (for signaling healthy), and found them attractive, but also reported that this made it more difficult to distinguish the FOPLs from the package. With regard to warning labels in particular, the use of the word “WARNING” and the exclamation mark was memorable and indicated danger, consistent with previous studies [30,31,75]. Some adolescents suggested these design elements be used for the red-labeled nutrients on the TLL as well. Taken together, these results suggest that if colors are used, design elements like a holding strip or white border should be used to ensure adequate contrast with product packaging, and that elements like “warning” are particularly useful to signal unhealthy.

This study had several strengths and limitations. Strength of this study is that it sought to test adolescents’ reactions to FOPL with a focus on specific steps on the conceptual pathway from FOPL exposure to behavioral change. However, at the same time, the study relied on self-reported perceptions, which may not completely predict behavioral responses. Sampling bias may have also affected the results, as individuals with certain characteristics might have been more inclined to participate in the study. In addition, participants evaluated labels on a small subset of products, which neither represents the wide array of products available in the Indonesian food supply nor the complex food retail environment in which adolescents make decisions. Although adolescents believed that FOPLs would not influence their snack purchase, the overall data on warning labels specifically show that they do change purchasing behavior [16,76,77], though very little research has been done in the adolescent population specifically. Thus, future experimental research would be useful in understanding how FOPLs impact adolescents’ food purchasing behaviors in a more realistic environment.

In conclusion, although TLLs are best liked among Indonesian adolescents, warning labels were perceived as most likely to help adolescents identify unhealthy foods and discourage their purchases. The “Healthy Choice” label was perceived as less effective. Although TLL were considered more attractive and more believable, warning labels were perceived as more attention-grabbing, more likely to make participants stop and think, and more likely to influence purchases. Future research is needed to understand whether FOPLs will impact adolescents’ food purchasing behaviors and whether there are differences by gender or SES. Additionally, enhancing the efficacy of FOPLs in real-life settings requires the identification of complementary interventions aimed at raising awareness and promoting the utilization of FOPLs. This is crucial for formulating a comprehensive policy action.

## Author contributions

The authors’ contributions were as follows – AS, ZF, NE, ADD: contributed to data collection and data analysis; LST, WG: performed data interpretation; LST: wrote the first draft; WG: edited the first draft; and all authors: contributed to the study design, read, and approved this manuscript.

## Data availability

Data described in the manuscript, code book, and analytic code will be made available upon request by emailing the corresponding author.

## Funding

This research was primarily funded by the Dutch Ministry of Foreign Affairs, Activity number 4000005271. This research was also supported by Bloomberg Philanthropies.

## Conflict of interest

The authors report no conflicts of interest.
