# Supplementary material for: Indonesian Adolescents’ Perceptions of Front-of-Package Labels on Packaged Food and Drinks
Source: Curr Dev Nutr. 2025 Mar 8;9(4):104586. doi: 10.1016/j.cdnut.2025.104586 (PMC12019014; doi:10.1016/j.cdnut.2025.104586)
Supplement: Multimedia component 1 [file mmc1.docx]

**Supplementary material 1. Focus group discussion guide:**

**1. Introduction and Explanation to Participants:**

“Hello, my name is……, I will be conducting the session today, and this is ……, who will assist me by taking notes and helping in the conduct of the research.

Thank you for participating in this study today. We’re are interested in how adolescents make decisions to buy foods. We are also interested in peoples’ reactions to different labels for the front of food and drink packages. I will show you different labels. I will ask you for your reactions to it.

This study is all about YOUR experiences. There are NO right or wrong answers to anything we discuss today. Having your honest opinion is very important. When you were invited to participate in this discussion today, you already received an information sheet explaining the purpose of the study as well as the measures that will be taken to ensure that your identify will not be revealed to anyone, and that you may stop participating at any time without any problems or consequences. Your identity will not be provided to anyone outside this research project. Your answers will be combined with the answers of others so that your identity cannot be known. Therefore, please answer all questions without hesitation. If any of you have questions about the project and your participation, I can answer those questions now. Please also feel free to stop me during the discussion if you think of another question.

As we are here as part of a group, you will hear information about the others in this room and you will also hear their opinion on the labels that we will show you. To give each one in this room the assurance that their identity and their opinions will not be shared by any of you outside of this room, we also need to agree with each other that we will not share information with others. In front of you, you will find a consent form stating all of this which you can sign as such an agreement to each other. “Does this sound okay to you? Shall we proceed? ” [*Moderator, please note that the consent form must be a loose page – not binded in the booklet with responses – and must be collected before participants proceed to filling in the forms, in order to signal separation of their names/signatures from their responses.]*

“With your permission, we would like to record the group. [*Moderator, if the groups are being viewed from a one-way mirror, or if they are being webcast, please inform the participants of this and assure them of the confidentiality of their participation.*] The recording will only be used to help us with analyzing the results. Your personal details are confidential, and we will not keep or pass on any personal information about you. Is it OK for us to record the group? Can each of you please sign the consent form to indicate your agreement.”

*[Turn the recording equipment on to record this part of the research – the recording will help with analyzing the results and key points from the discussion.]*

[*Moderator: Ensure that the socio-demographic questionnaires and rating sheets are available in the language of the participants. Ensure that all participants have completed the demographic questionnaire on page #2 of the booklet before commencing. If some have not done so yet, then allow them to finish it here before you begin*.]

**Warm-up: Participants’ introduction**

Before we go on to the discussion about food purchasing decisions, it would help us to know a little bit about each of you and for us to get to know one another. Please tell us your name and your favorite movie, song, or book from the last year ... I’ll start ….

*[NOTE to moderator: The goal here is to introduce each person and make everyone comfortable with one another.]*

**Section 1. Discussion about food purchases**

Now, I am going to show you an image of some products and then we will discuss what you see. First, I want to make sure that you can all see the images that I project clearly. [Show the initial set of general products and confirm participants can see them].

Now, I am going to ask you some general questions about how you make food purchasing decisions.

Please proceed to complete the rating exercise. Please do so silently and independently. You may ask me if you have any questions or doubts about this task.

*Note, for this* ***and all such rating exercises*** *within this guide, with low literacy groups, an assistant should be available to help participants complete the rating sheet. In this case, the moderator should read each question out loud, ensure that every participant has completed it – with assistance as required – and then proceed to the next question until all questions in the rating sheet have been completed.]*

Once everyone has completed the rating exercise, begin the general discussion.

**General Discussion**

1. When you are selecting a packaged food or drink at the store, what are the most important factors in your decision to buy it?
2. How often do you use nutrition labels on the BACK of food and drink packages to guide your purchasing decisions?
   1. What information do you typically look for? What information on packages either increases or decreases your likelihood of purchasing a certain food or drink?
3. How often do you use nutrition labels on the FRONT of food and drink packages to guide your purchasing decisions?
   1. What information do you typically look for? What information on packages either increases or decreases your likelihood of purchasing a certain food or drink?
4. Do you look for claims about the healthfulness of foods and drinks? What particular health claim do you look for/ guides your decision to purchase a food or drink? [*Moderator, probe whether participants look at ingredients, nutrition labels, warning labels, health claims, brands etc*.]
5. What makes a product packaging appealing to you? What draws you to a specific product? What sorts of images are appealing? [*Moderator, probe for celebrities, cartoons, sports figures, or other features*]
6. How much does the price of a product influence your decision of what to buy?
7. How much do you consider whether a food is healthy or unhealthy?
8. How much do you consider whether a food is considered “cool”?
9. If you saw a label on a food or drink product telling you the food is healthy or unhealthy, what would it mean to you if you saw a government endorsement of the label? For example, from the Ministry of Health? [*Moderator, probe for importance*]
10. Are there important factors that make you want to buy a food or drink that we haven’t already discussed?

**Section 2. FOPL Testing**

**Optional 2A. Example Label rating**: *show participants set of 4 general products again, perhaps with a neutral icon or label (for example- could use India’s V for Vegetarian label) and have them complete an example rating exercise (using the same rating items as in the Main Label Testing), the purpose of this would be to have them practice viewing and rating the label.*

**Section 2B. Main Label Testing:**

“Now, I’m going to show you all an image and then ask you some questions about it.”

*Then, proceed to show the main image for testing Label #1 (e.g., the 4 products with Label #1). Keep it up for 10 seconds or so until everyone seems to have seen it clearly. Then turn off the image.*

Visibility/ Memorability

- Did you notice any labels on the packages? Before we discuss it together, in the questionnaire before you, please turn to page #X [*Moderator, direct participants to the appropriate place in the* booklet] and either draw or describe – whatever you prefer – what you recall seeing. Don’t talk with one another as you do this, please recall this independently and then we can discuss it together. [*Moderator, please give participants about 3 minutes to complete this.*].
- Now, I’d like to know what you understood from the label(s)? What was or were the main messages? Please explain this in the next question on page #X [*Moderator, direct participants to the space provided for this in the rating sheet.]*

“Now, I’m going to project the image back up again and this time I want you to focus closely on a set of labels you will see on the front of the food and drink packages. Study this set of labels closely and I will ask you some questions about it.”

[*Moderator: Project the image of Label #1 back again for about 10 seconds. Point to the FoPLs and ensure that all participants have seen them clearly. Once all participants have seen them clearly, turn off the image.]*

Now, please use your form answer the questions there about the labels. Please know that there are no right or wrong answers. We want to know what you think. Hence, please answer the questions independently and without consulting your neighbour. We will have a chance to discuss your answers together later.

[*Moderator, once the rating has concluded, please ask them to close the questionnaire and proceed to the discussion.]*

*[If necessary, reiterate the following: I would like to reiterate that your experience is really important and there is no right and wrong answers. Also, as we are talking about your personal opinions and experiences, it is not necessary for everyone to agree with each other. It is helpful for us to find out the different opinions that people have, as well as where people have the same opinions, so please feel free to tell us whatever you think and feel, even if it might be different to what other people in the room are saying. Also, let’s please make sure that only one person speaks at a time. Please allow each person to complete what they are saying.]*

Visibility/ Memorability

Were the labels easily visible? Did it grab your attention? How visible was it? Was it immediately visible or not? Did it catch your eye?

Were the labels memorable?

Can you recall the label for me now? What exactly did it look like? What do you recall of its shape, colour? Was there any text in it? What did it say? [*Without leading their answers, probe respondents’ memory of its shape, colour, text, icon etc.]*

Comprehensibility

What do you think is the purpose of these labels?

What did you understand from the labels?

Is there anything you did not understand about the label or that confused you about it?

What did the labels tell you about the food and drinks they were on? (probe for if they thought the product was healthy or unhealthy)

Who do you think these labels are for?

Did you believe what the label said?

Is there anything about this label that is culturally inappropriate? Is there anything about it that is likely to be difficult to understand/interpret for other teenagers like you in Indonesia?

Potential Effectiveness

If you were at the store and saw these labels on a food or drink packages, would they change your attitude toward the product? How?

If you saw this label on food or drink packages in a store, would it affect your decision to buy that product or not? *How* would it affect your decision to buy it?

Would you use this label more for new products, that you never had before, or products that you often buy?

In your opinion, what would be the benefits of placing labels like this on unhealthy foods? Whom will they help?

In your opinion, what are the harms of placing labels like this on unhealthy foods? Whom would they hurt?

How do you think other Indonesian teenagers would react if they saw these labels on food and drink products in stores?

What impact do you think these labels would have on Indonesian teenagers?

Do you think these labels would affect other adolescents’ purchasing decisions? If so, how?

Improvements to the label’s memorability

What aspect of the label had the most impact on you and why?

What was most MEMORABLE part of these labels? What image do you remember most? What words do you remember most?

**Section 2C. Comparative Rating**

*Show a slide that contains all three label types (Warning, GDA, and Healthy Icon).*  Now please look at all three labels you have seen today. Please turn to page [xx] and complete the questions there.

Now, thinking of all the labels you’ve seen today, does any **one** label stand out for you? Which one

would that be? What about that label makes it stand out the most? Why do the other labels not stand out as much to you?

Which label do you think would most help you you identify identify that a food was unhealthy? Which label would most discourage you from buying unhealthy foods?

**Conclusion**

Thank participants for their time and conclude.

**Appendix B. Label Rating Exercise**

Instructions. Considering the LABEL you just saw, please tell me how much you agree or disagree with each statement.

| **This label…** | **Strongly disagree** | **Somewhat disagree** | **Neither disagree nor agree** | **Somewhat agree** | **Strongly agree** |
| --- | --- | --- | --- | --- | --- |
| … is easy to see | - **1** | - **2** | - **3** | - **4** | - **5** |
| … is memorable | - **1** | - **2** | - **3** | - **4** | - **5** |
| …is believable | - **1** | - **2** | - **3** | - **4** | - **5** |
| …makes me stop and think | - **1** | - **2** | - **3** | - **4** | - **5** |
| …is relevant | - **1** | - **2** | - **3** | - **4** | - **5** |
| …helps me know if which foods/drinks are unhealthy | - **1** | - **2** | - **3** | - **4** | - **5** |
| ……makes me concerned about purchasing unhealthy foods/drinks | - **1** | - **2** | - **3** | - **4** | - **5** |
| …… makes me not want to buy foods and drinks that are unhealthy | - **1** | - **2** | - **3** | - **4** | - **5** |
| I would be less likely to purchase unhealthy food/drink if I saw this label on products | - **1** | - **2** | - **3** | - **4** | - **5** |

**Appendix C. Comparison between labels**

1. Of the three options [FOPL], which one is more likely to grab your attention? (Check one only)

- Option 1
- Option 2
- Option 3
- All three are about the same

1. Of the three options shown on the screen, which one will help you tell what foods and drinks are unhealthy (Check one only)

- Option 1
- Option 2
- Option 3
- All three are about the same

1. Of the three options shown on the screen, which one is more likely to make you avoid purchasing unhealthy food or drink? (Check one only)

- Option 1
- Option 2
- Option 3
- All three are about the same
